# In Response to: “Using Lean-Based Systems Engineering to Increase Capacity in the Emergency Department

**DOI:** 10.5811/westjem.2014.11.24355

**Published:** 2014-12-15

**Authors:** Marian J. Vermeulen, Michael J Schull

**Affiliations:** Institute for Clinical Evaluative Sciences, Canada

White BA, Chang Y, Grabowski B G. Using Lean-Based Systems Engineering to Increase Capacity in the Emergency Department. *West J Emerg Med.* 2014;15(7):770–776.

To the editor:

We read with interest the article by White et al., “Using Lean-Based Systems Engineering to Increase Capacity in the Emergency Department,” in which the authors conclude that Lean could improve emergency department (ED) throughput and capacity.

A number of other studies have also suggested that Lean is beneficial in addressing the problem of ED wait times. As in White et al., the vast majority of these studies have been conducted in single centers and/or as before-after evaluations.^1^–^6^ Moreover, publication bias likely also plays a role in the consistency of these findings since positive evaluations are more likely to be published.^7^ Although White et al. compared changes in ED length of stay with a concurrent population in their own center, it is not possible to generalize beyond this particular ED.

We recently published a large multi-center controlled study of Lean in Ontario, Canada, (http://www.annemergmed.com/article/S0196-0644%2814%2900516-2/fulltext) and found that while there were reductions in ED length of stay among the 36 hospitals that participated in the Lean program, similar reductions were observed among the 63 matched control hospitals over the same period. In our study, context was also important. Because Lean was part of a broader ED wait time strategy, including wait time targets, public reporting, and targeted financial incentives, it was clear that a wide array of incentives had an effect on wait times in all EDs across the region.

Our conclusion is that single-center and before-after studies do not provide rigorous or generalizable evidence that Lean is effective in reducing ED length of stay. Decisions to implement should be based on solid evidence, since Lean initiatives typically require the engagement of external consultants and/or the dedication of significant internal resources for their development and implementation.

## References

[1] Alikhan LM, Howard RJ, Bowry R (2009). From a project to transformation: how “going against the flow” led to improved access and patient flow in an academic hospital. Healthc Manage Forum.

[2] Ford AL, Williams JA, Spencer M (2012). Reducing door-to-needle times using Toyota’s lean manufacturing principles and value stream analysis. Stroke.

[3] Mazzocato P, Holden RJ, Brommels M (2012). How does lean work in emergency care? A case study of a lean-inspired intervention at the Astrid Lindgren Children’s hospital, Stockholm, Sweden. BMC Health Serv Res.

[4] Murrell KL, Offerman SR, Kauffman MB (2011). Applying lean: implementation of a rapid triage and treatment system. West J Emerg Med.

[5] Ng D, Vail G, Thomas S, Schmidt N (2010). Applying the Lean principles of the Toyota Production System to reduce wait times in the emergency department. CJEM.

[6] Piggott Z, Weldon E, Strome T (2011). Application of Lean principles to improve early cardiac care in the emergency department. CJEM.

[7] Song F, Parekh S, Hooper L (2010). Dissemination and publication of research findings: an updated review of related biases. Health Technol Assess.

